# A Secure and Efficient Scalable Secret Image Sharing Scheme with Flexible Shadow Sizes

**DOI:** 10.1371/journal.pone.0168674

**Published:** 2017-01-10

**Authors:** Dong Xie, Lixiang Li, Haipeng Peng, Yixian Yang

**Affiliations:** 1 Information Security Center, State Key Laboratory of Networking and Switching Technology, Beijing University of Posts and Telecommunications, Beijing, 100876, China; 2 School of Mathematics and Computer Science, Anhui Normal University, Wuhu, 241000, China; 3 National Engineering Laboratory for Disaster Backup and Recovery, Beijing University of Posts and Telecommunications, Beijing, 100876, China; 4 State Key Laboratory of Public Big Data, Guizhou, 550025,China; Kaohsiung Medical University, TAIWAN

## Abstract

In a general (*k*, *n*) scalable secret image sharing (SSIS) scheme, the secret image is shared by *n* participants and any *k* or more than *k* participants have the ability to reconstruct it. The scalability means that the amount of information in the reconstructed image scales in proportion to the number of the participants. In most existing SSIS schemes, the size of each image shadow is relatively large and the dealer does not has a flexible control strategy to adjust it to meet the demand of differen applications. Besides, almost all existing SSIS schemes are not applicable under noise circumstances. To address these deficiencies, in this paper we present a novel SSIS scheme based on a brand-new technique, called compressed sensing, which has been widely used in many fields such as image processing, wireless communication and medical imaging. Our scheme has the property of flexibility, which means that the dealer can achieve a compromise between the size of each shadow and the quality of the reconstructed image. In addition, our scheme has many other advantages, including smooth scalability, noise-resilient capability, and high security. The experimental results and the comparison with similar works demonstrate the feasibility and superiority of our scheme.

## Introduction

To safeguard cryptographic keys, Shamir [1] and Blakley [2] presented a novel method for constructing secure and robust key management schemes, called secret sharing (SS), which can overcome many vulnerabilities when one possesses a single master key for a certain cryptosystem. From a cryptographic perspective, SS scheme refers to some methods for distributing a secret among a group of participants and each one in the group is allocated a share or shadow of the secret. The secret can be reconstructed only when a sufficient number of shadows are combined together and individual shadow serves no useful purpose for obtaining it. As a secure and fault-tolerant technique, SS has beed widely used in many fields, such as distributed storage system, secure multiparty computation, and electronic voting [3, 4]. In Shamir’s (*k*, *n*)(2 ≤ *k* ≤ *n*) threshold SS scheme [1], the secret is divided into *n* shadows and any *k* of them can reconstruct the whole original secret. However, any less than *k* shadows cannot obtain any information about the secret. Although this construction method is information theoretically secure, each participant requires relatively large storage space because the size of each shadow is equal to that of the secret data. Therefore, using Shamir’s SS scheme will result in huge communication burden when we share image or video data at the pixel level. In fact, the topic of designing SS scheme for image data has attracted wide attention in the past fifteen years.

In 2002, Thien and Lin [5] proposed a (*k*, *n*) secret image sharing (SIS) scheme based on Shamir’s (*k*, *n*) threshold SS scheme. In their scheme, the dealer first generates a *k* − 1 degree polynomial by letting the *k* coefficients be the gray values of *k* pixels in the image and then computes the corresponding shadow for each participant according to the polynomial. In fact, the main difference between their method and Shamir’s scheme is that they use no random coefficient. In 2004, Lin and Tsai [6] proposed a SIS scheme with the additional capabilities of steganography and authentication. Unfortunately, dishonest participants in their scheme can easily manipulate the stego-image for successful authentication but cannot recover the secret image. Subsequently, Yang et al. [7] presented an improved scheme to enhance authentication ability that prevents dishonest participants from cheating. In 2010, Alharthi and Atrey [8] specifically analyzed the SIS scheme presented by Thien and Lin [5], and proposed an improved scheme for secure image sharing. In addition, there are some other related research results about this topic in recent years, e.g., [9–11].

In a (*k*, *n*) threshold SIS scheme, the secret image can be completely reconstructed if more than *k* shadows are available and any *k* − 1 or less shadows reveal no information about the image. However, this type of reconstruction policy that reveals either the entire image or nothing limits its possible applications. In some circumstances, it may require the secret image to be gradually reconstructed, which means that the clarity or quality of the reconstructed image depend on the number or characteristics of shadows participating in the reconstruction process. To this end, Wang and Shyu [12] first proposed the concept of scalable secret image sharing (SSIS), where single shadow reveals no information about the secret image and any *k*(2 ≤ *k* ≤ *n*) shadows can reconstruct the secret image in a scalable manner. In their scheme, the secret image is first divided into *n* disjoint sub images and generate two sub-share images using Thein and Lin’s (2,2)-SIS scheme. Then the sub-share images are encoded to produce shadow images. The main drawback of their proposed scheme is that the threshold value *k* is limited to 2. In 2010, Yang and Huang generalized Wang and Shyu’s (2, *n*)-SSIS scheme and proposed a general (*k*, *n*)-SSIS scheme [13]. Subsequently, Yang and Chu [14] presented a (*k*, *n*)-SSIS scheme with the smooth scalability, which means that the information amount of reconstructed image is “smoothly” proportional to the number of shadows used in reconstruction. To put it another way, the more the number of available shadows, the better the quality of the reconstructed image. In 2015, Liu et al. introduced a lossy SSIS method for color images [15], which has relatively small shadow size and also achieved good noise-resilient capability. In the same year, Lee and Chen developed a new method for constructing SSIS schemes based on the Salient map [16], in which the region of interesting of the secret image can be revealed progressively.

From a high-level perspective, the reconstruction phase in Shamir-type (*k*, *n*)-SIS schemes [1, 5, 8, 12] can be regarded as the process of solving the secret image **I** from **Y** = **AI** generated by the available shadows of participants, where $A\in R^{k_{1}\times k}$ is the known Vandermonde coefficient matrix and *k*_1_ denotes the number of available participants. As long as *k*_1_ is not smaller than *k*, the reconstruction can be performed by solving the aforementioned linear equations because the determinant of **A** is not equal to 0. Previous studies indicate that a natural image has high correlativity between pixels in one local region and it can be represented using only a few non-zero coefficients in a suitable basis or dictionary [17, 18]. However, to the best of our knowledge, almost all previous SIS schemes do not take full advantage of this sparsity, which may play an important role in reducing the size of each shadow.

In this paper, we present a secure and efficient (*k*, *n*)-SISS with flexible shadow sizes based on compressed sensing (CS) [19–21], which has attracted considerable attention over the last ten years because it can reconstruct sparse signals using a very few measurements. Employing the sparsity of image data, we can reconstruct the secret image **I** from relatively short available shadows. To reduce the size of each participant’s shadow as much as possible, we first sparsify the secret image and then compress it by chaotic sensing matrix in CS. After that, the obtained image is cut in a right-to-left fashion using a parameter *λ* which controls the size of each shadow. Subsequently, we divide the downsized image into *n* equal parts and perform two nonlinear operations on each part, including permutation and diffusion. Hence all shadows of the secret image **I** are formed. Roughly speaking, our proposed scheme in fact consists of two major steps, linear transformations (CS and downsizing) and nonlinear operations (permutation and diffusion). The linear procedures are used for decreasing the size of each participant’s shadow, and the nonlinear operations are used for improving security. In general, the advantages of our scheme can be summarized as follows:

- *High security*. The key space in our original scheme is large enough to make brute-force attacks infeasible and it can also resist ciphertext-only attack and known-plaintext attack. In addition, the generated shadows has small correlation coefficients of adjacent pixels because of linear transformations and nonlinear operations. Therefore, our construction provides a high-level security.
- *High efficiency*. In our scheme, the dealer needs to perform two important procedures, i.e., compression and downsizing. These two procedures play an important role in reducing the size of each shadow. The experimental results indicate the superiority of our scheme.
- *Noise-resilient capability*. In the real-world applications, noise exists everywhere. Unfortunately, most existing SSIS schemes are not applicable under noise circumstances. With the help of CS, our SSIS scheme can still work when there exist some types of noise in the communication channel.
- *Flexibility*. To the best of our knowledge, our proposed scheme is the first SSIS scheme with flexible shadows. The size of each shadow in our scheme can be controlled by a parameter. Thus it achieves a compromise between the size of each shadow and the quality of the reconstructed image.

The rest of this paper is organized as follows. Section 2 recalls some basic background, including Shamir’s (*k*, *n*)-SS scheme and CS. We describe our main idea in Section 3 and introduce our proposed scheme explicitly in Section 4. The experimental results and security analysis will be shown in Section 5. The comparison with similar works is described in Section 6. Finally, we conclude this paper in Section 7.

## 1 Background Knowledge

For any positive integer *n*, [*n*] denotes the set {1, 2, ⋯, *n*}. Throughout this paper, all vectors are assumed to be in column form and are written using bold lower-case letters (e.g. **a**). We use bold capital-case letters (e.g. **A**) to represent matrices. For a matrix **A**, the vectorization of **A**, denoted by *vec*(**A**), is a long column vector obtained by stacking the columns of **A** on top of one another. Given two functions *f* and *g* defined on some subset of the real numbers, *f*(*x*) = *O*(*g*(*x*)) if and only if there is a positive constant *C* and a real number *x*_0_ such that |*f*(*x*)| ≤ *C*|*g*(*x*)| for all *x* ≥ *x*_0_.

### 1.1 Shamir’s (*k*, *n*)-SS scheme

Based on polynomial interpolation, Shamir presented a (*k*, *n*)-SS scheme [1] in 1979, which has been widely used in various practical applications. In his scheme, the dealer shares a secret *a*_0_ into *n* parts for *n* participants by computing a random *k* − 1 degree polynomial. The reconstruction phase requires at least *k* participants and with even *k* − 1 or less, recovery is impossible. In detail, the dealer first chooses a large prime number *p* (*p* > *a*_0_) and *k* − 1 integers {*a*_*i*_}_*i*∈[*k*−1]_ such that each integer is smaller than *p*. Therefore, the random *k* − 1 degree polynomial is formed as:

$$f\left(x\right)=a_{0}+a_{1}x+a_{2}x^{2}+\cdots +a_{k-1}x^{k-1}\;\mathrm{mod}\;p.$$ (1)

The dealer then computes the *i*-th shadow *y*_*i*_ = *f*(*i*) for the *i*-th participant, where 1 ≤ *i* ≤ *n*. Using Lagrange interpolation, any *k* or more than *k* shadows can reconstruct the secret *a*_0_ through

$$a_{0}=f\left(0\right)=\sum_{i=1}^{k}y_{i}\prod_{j=1,j\neq i}^{k}\frac{-j}{i-j}\;\mathrm{mod}\;p.$$ (2)

### 1.2 Compressed Sensing

The theory of CS [19–22] breaks the limitation of Nyquist-Shannon sampling theorem because it can reconstruct sparse signals using a very few measurements. It has attracted considerable attention and been widely used in many fields such as image processing, wireless communication, medical imaging, and identification problem [23]. The standard model of CS is an underdetermined linear system defined as follows,

y=Ax, (3)

where $A\in R^{M\times N}$, $x\in R^{N}$, $y\in R^{M}$ and *M* < *N*. From a signal processing perspective, **A**, **x** and **y**, respectively, represent the sensing matrix, the signal vector, and the compressed measurements.

To recover the original signal **x** from the compressed measurements **y**, it requires that **x** is sparse and the sensing matrix **A** must satisfy some special properties. We say a vector **x** is *s*-sparse if it has at most *s* non-zero entries. Previous studies showed that if the sensing matrix **A** satisfies the Restricted Isometry Property (RIP) [19–21], then the sparse signal **x** can be exactly recovered with overwhelming probability. Next, we will recall the condition for uniqueness of sparse solutions to Eq (3) and the definition of the RIP.

**Definition 1** ([19]) *The spark of a given matrix* **A** *is the smallest number of columns of* **A** *that are linearly dependent*.

**Lemma 1** ([24]) *For any vector* $y\in R^{M}$, *there exists at most one s-sparse signal* **x** *such that* **y** = **Ax** *if and only if spark*(**A**) > 2*s*.

**Definition 2** *A matrix* **A** *satisfies the restricted isometry property (RIP) of order s if there exists a* *δ*_*s*_ ∈ (0, 1) *such that*

$$\left(1-\delta_{s}\right){∥v∥}_{2}^{2}\;\leq \;{∥\text{Av}∥}_{2}^{2}\;\leq \;\left(1+\delta_{s}\right){∥v∥}_{2}^{2},$$ (4)

*holds for all s-sparse vectors* **v**.

Given the sensing matrix **A**, there are a great variety of algorithms to recover the original signal **x** efficiently, such as the classical greedy Orthogonal Matching Pursuit (OMP) algorithm [25] and *l*_1_ optimization algorithm [26]. We use the OMP algorithm in our experiments.

To reduce storage space of sensing matrices, Yu et al. [27] proposed a novel method for constructing sensing matrices using chaotic (logistic map) sequences, and also proved that it satisfy the RIP with overwhelming probability if the sparsity *s* ≤ *O*(*M* / log(*N*/*s*)). However, the probability density function of logistic map does not follow the uniform distribution in the interval (0, 1). In order to enhance the security, it is necessary to select a chaotic map which can produce pseudo random sequences. Frunzete et al. [28] proposed to construct sensing matrices using the one-dimensional skew tent map, whose probability density function follows the uniform distribution. Specifically, the skew tent map with parameter *μ* is given by

$$z\left(n+1\right)=\left\{\begin{array}{ccc} \frac{z(n)}{\mu } & & 0<z(n)<\mu \\ \frac{1-z(n)}{1-\mu } & & \mu \leq z(n)<1 \end{array}\right.,$$ (5)

where *z*(0) ∈ (0, 1) is the initial value and *μ* ∈ (0, 1) is a parameter.

**Lemma 2** ([27, 28]) *Chaotic sensing matrix* $A\in R^{M\times N}$ *constructed by the logistic map or the skew tent map satisfies the RIP for constant* *δ*_*s*_ > 0 *with overwhelming probability providing that s* ≤ *O*(*M/* log(*N/s*)).

## 2 Main idea

All arithmetic operations in Shamir’s (*k*, *n*)-SS scheme [1] are performed over the finite field $Z_{p}^{*}$. Naturally, the proposed method can be directly extended to the real number field R. We assume that these two cases are not to be distinguished throughout this paper. For *i* = 1, ⋯, *n*, let *P*_*i*_ denote the *i*-th participant. From a linear algebra point of view, the sharing phase can be viewed as a problem of computing the following linear equations:

$$\left(\begin{array}{cccc} 1 & 1 & \cdots & 1\\ 1 & 2 & \cdots & 2^{k-1}\\ \vdots & \vdots & \vdots & \vdots \\ 1 & k & \cdots & k^{k-1}\\ \vdots & \vdots & \vdots & \vdots \\ 1 & n & \cdots & n^{k-1} \end{array}\right)\left(\begin{array}{c} a_{0}\\ a_{1}\\ \vdots \\ a_{k-1} \end{array}\right)=\left(\begin{array}{c} y_{1}\\ y_{2}\\ \vdots \\ y_{k}\\ \vdots \\ y_{n} \end{array}\right).$$ (6)

Support that the *k* participants {*P*_*i*_*j*__}_*j* ∈ [*k*]_ want to reconstruct the secret *a*_0_, then the reconstruction phase can be viewed as a problem of solving the following linear equations:

$$\left(\begin{array}{cccc} 1 & {i_{1}}^{1} & \cdots & {i_{1}}^{k-1}\\ 1 & {i_{2}}^{1} & \cdots & {i_{2}}^{k-1}\\ \vdots & \vdots & \vdots & \vdots \\ 1 & {i_{k}}^{1} & \cdots & {i_{k}}^{k-1} \end{array}\right)\left(\begin{array}{c} a_{0}\\ a_{1}\\ \vdots \\ a_{k-1} \end{array}\right)=\left(\begin{array}{c} y_{i_{1}}\\ y_{i_{2}}\\ \vdots \\ y_{i_{k}} \end{array}\right).$$ (7)

Let $A=\left(\begin{array}{cccc} 1 & i_{1}{}^{1} & \cdots & i_{1}{}^{k-1}\\ 1 & i_{2}{}^{1} & \cdots & i_{2}{}^{k-1}\\ \vdots & \vdots & \vdots & \vdots \\ 1 & i_{k}{}^{1} & \cdots & i_{k}{}^{k-1} \end{array}\right)$, $a=\left(\begin{array}{c} a_{0}\\ a_{1}\\ \vdots \\ a_{k-1} \end{array}\right)$, and $y=\left(\begin{array}{c} y_{i_{1}}\\ y_{i_{2}}\\ \vdots \\ y_{i_{k}} \end{array}\right)$. Note that **A** is a *k* × *k* Vandermonde matrix and its determinant is not equal to 0. Thus the vector **a** containing the secret *a*_0_ can be obtained from the vector **y**, which is formed by *k* shadows {**y**_*i*_*j*__}_*j*∈[*k*]_. This method is not suitable for sharing image data at the pixel level because the size of each shadow is at least the same as the size of the secret image. To overcome this disadvantage, Thien and Lin introduced a variant [5], where the gray values of *k* pixels in the secret image are set to all entries of **a**. Therefore the size of each shadow can be reduced by a factor of *k*.

From Eqs (3) and (7), we can see that the model of CS is very similar to that of Shamir-type (*k*, *n*)-SIS schemes. It is worth mentioning that CS possesses the characteristic of compression, but Shamir-type schemes do not. Thus, we try to use CS to replace the method of Shamir-type schemes for sharing secret images. In fact, the compression and sensing process in CS can be regarded as the sharing and reconstruction phase in (*k*, *n*)-SIS schemes respectively. Fortunately, some image data can be represented using only a few non-zero coefficients in a suitable basis or dictionary [17, 18]. That is, the coefficient vector **a** can be viewed as a sparse vector. This meets the sparsity condition in CS. However, the formed coefficient matrix **A** is a Vandermonde matrix and it can not be used as a sensing matrix because the recovery process of CS is numerically unstable [29]. In CS, some chaotic systems can be used to produce sensing matrices because the sensing device only requires to store some private initial values. More importantly, Lemma 2 shows that sensing matrix **A** constructed by the logistic map or the skew tent map satisfies the RIP with overwhelming probability providing that *s* ≤ *O*(*M* / log(*M*/*s*)). Therefore, the dealer can use a chaotic system to produce a lower dimensional matrix **A** and compress the secret image. It is not hard to see that the reconstruction phase can be performed evidently using some reconstruction algorithms in CS. To improve the security of our scheme, we need two additional nonlinear transformations, including permutation and diffusion. The experimental results and the comparison with similar works indicate the feasibility and potential superiority of our scheme.

## 3 Our Proposed Scheme

**Fig 1:**
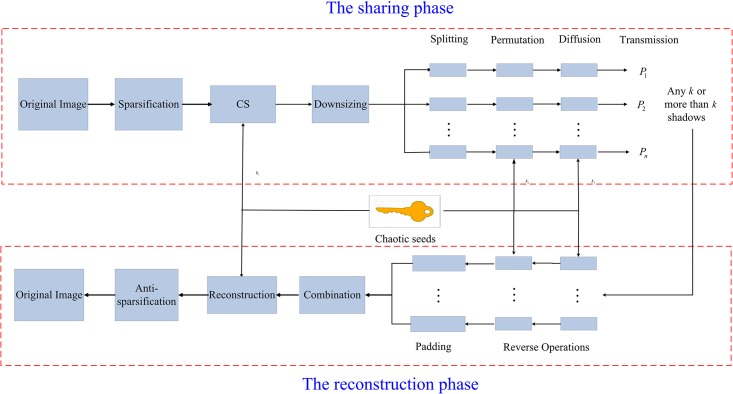
Schematic diagram of our proposed scheme.

In this section, we will describe our proposed SSIS scheme in detail. Fig 1 shows the schematic diagram of the proposed method. Similar to some previous related schemes, we assume that there is a trusted key distribution center in our scheme, which distributes private keys *k*_1_, *k*_2_ and *k*_3_ for the dealer and the *n* participants over a secure channel. This section consists of three parts: the secret image sharing phase, the image reconstruction phase and several theoretical results.

### 3.1 The secret image sharing phase

Suppose that the dealer wants to divide the secret image **I** ∈ {0, 255}^*N* × *N*^ into *n* smaller shadows **I**_1_, ⋯, **I**_*n*_. Now we describe the secret image sharing phase of our SSIS scheme as follows:

1. *Sparsification*. Choose the discrete wavelet transformation (DWT) to represent the secret image **I** and obtain the corresponding sparse matrix $X\in R^{N\times N}$, i.e., **X** = **ΦIΦ**^*T*^, where **Φ** is the *N*-dimensional orthogonal DWT matrix.
2. *Compression*. Based on the construction method for sensing matrices [28], generate the sensing matrix $A\in R^{M\times N}$ using the private key *k*_1_ = (*z*^1^(0), *μ*_1_), where *M* is a multiple of *n*. Then compress the matrix **X** and obtain $Y\in R^{M\times N}$, i.e., **Y** = **AX**.
3. *Downsizing*. To reduce the size of each shadow, cut **Y** in a right-to-left fashion through a control parameter *λ* ∈ (0, 1] such that λN∈Z and obtain a tailored matrix $Z\in R^{M\times \lambda N}$.
  $$Y=\left(\begin{array}{ccccc} y_{1,1} & \cdots & y_{1,\lambda N} & \cdots & y_{1,N}\\ y_{2,1} & \cdots & y_{2,\lambda N} & \cdots & y_{2,N}\\ \vdots & \vdots & \vdots & \vdots & \vdots \\ y_{M,1} & \cdots & y_{M,\lambda N} & \cdots & y_{M,N} \end{array}\right)\overset{\text{downsizing}}{\Rightarrow }Z=\left(\begin{array}{ccc} y_{1,1} & \cdots & y_{1,\lambda N}\\ y_{2,1} & \cdots & y_{2,\lambda N}\\ \vdots & \vdots & \vdots \\ y_{M,1} & \cdots & y_{M,\lambda N} \end{array}\right).$$ (8)
4. *Splitting*. Split **Z** into *n* matrices {**Z**_*i*_}_*i*∈[*n*]_ with the same size, i.e.,
  $$Z=\left(\begin{array}{c} Z_{1}\\ \vdots \\ Z_{n} \end{array}\right),$$ (9)
  where $Z_{i}\in R^{M/n\times \lambda N}$.
5. *Permutation*. In order to improve the security, the permutation method proposed by Wang et al. [30] has been widely used in some image encryption systems. Here we use a slight variant to perform this step.
  - Use the private key *k*_2_ = (*z*^2^(0), *μ*_2_) to iterate the skew tent map *m* + *λMN*/*n* times and discard the first *m* values to obtain the sequence ${\left\{z\left(i\right)\right\}}_{i=m+1}^{m+\lambda MN/n}$. Note that the reason we discard the first *m* values is the transient effect.
  - Sort the remaining *λMN*/*n* values to obtain a new sequence ${\left\{\tilde{z}\left(i\right)\right\}}_{i=m+1}^{m+\lambda MN/n}$.
  - Search the values of ${\left\{z\left(i\right)\right\}}_{i=m+1}^{m+\lambda MN/n}$ in ${\left\{\tilde{z}\left(i\right)\right\}}_{i=m+1}^{m+\lambda MN/n}$, and form the corresponding indices ${\left\{Ind\left(i\right)\right\}}_{i=1}^{\lambda MN/n}$.
  - Perform the permutation procedure according to the indices ${\left\{Ind\left(i\right)\right\}}_{i=1}^{\lambda MN/n}$. For each *i*, let **q**_*i*_ = *vec*(**Z**_*i*_) and
    $$q_{i}^{*}=\left(\begin{array}{c} q_{i}\left(Ind\left(1\right)\right)\\ \vdots \\ q_{i}\left(Ind\left(\frac{\lambda MN}{n}\right)\right) \end{array}\right)\in R^{\frac{\lambda MN}{n}}.$$ (10)
  Reshape $q_{i}^{*}$ (i.e., anti-vectorization operation) and obtain $Z_{i}^{*}\in R^{M/n\times \lambda N}$.
6. *Diffusion*. Since permutation cannot change the statistical properties of the image, we need this step to resist some statistical attacks.
  - Use the private key *k*_3_ = (*z*^3^(0), *μ*_3_) to produce a sequence with *m* + *λMN*/*n* values using the skew tent map and then discard the first *m* values.
  - Reshape the remaining *λMN*/*n* values and obtain a matrix $R\in R^{M/n\times \lambda N}$.
  - Compute $R^{*}=10^{15}\cdot R\;\mathrm{mod}\;256$ and $I_{i}=Z_{i}^{*}+R^{*}$ for each *i*.
7. *Transmission*. Each image shadow **I**_*i*_ is distributed to the *i*-th participant *P*_*i*_.

**Remark 1** *Although CS is a simple linear transformation, we can view it as a symmetric encryption system whose private key is the sensing matrix. The security properties of this model have been studied in recent years, and the results show that it can provide computational security and resist some cryptographic attacks. In order to further improve the security of our scheme, we add two additional nonlinear steps, permutation and diffusion, which are widely used in classical image encryption schemes*.

**Remark 2** *In order to reduce the size of each shadow, we cut the compressed measurements* **Y** *and take the left part of it thought a control parameter λ in the fourth step of our scheme. In fact, if λ* = 1, *then the downsizing step disappears. Note that DWT provides a compact representation of a image in time and frequency that can be computed efficiently. It means that DWT has good properties of time-frequency localization and energy concentration. Thus, the most of the original image information are concentrated on the top-left corner of the image after sparsification and the left part of the image after CS. Thus the downsizing step is reasonable and the later experiments provide evidences for this claim*.

### 3.2 The image reconstruction phase

Without loss of generality, suppose that the *k* participants {*P*_*i*_}_*i*∈[*k*]_ with *k* shadows {**I**_*i*_}_*i* ∈ [*k*]_ want to reconstruct the secret image **I**. Then the image reconstruction phase can be performed as follows:

1. *Reverse operations*. For each *i*, perform the reverse operations of diffusion and permutation over **I**_*i*_ using the private keys *k*_3_ and *k*_2_. Therefore obtain **Z**_*i*_. Note that according to the secret sharing phase, the reverse operations can be implemented efficiently.
2. *Padding*. For each *i*, pad a **0** matrix with *M/n* × (1 − λ)*N* size on the right side of **Z**_*i*_ and form a new matrix $Y_{i}\in R^{M/n\times N}$.
3. *Extracting*. Generate the sensing matrix **A** using the private key *k*_1_ and extract the *i*-th part **A**_*i*_ of **A**, i.e.,
  $$A=\left(\begin{array}{c} A_{1}\\ \vdots \\ A_{n} \end{array}\right),$$ (11)
  where $A_{i}\in R^{M/n\times N}$.
4. *Combination*. Combine {**A**_*i*_}_*i* ∈ [*k*]_ and form a new sensing matrix $A^{*}\in R^{kM/n\times N}$. Similarly, form the compressed measurements $Y\in R^{kM/n\times N}$ using {**Y**_*i*_}_*i* ∈ [*k*]_.
5. *Reconstruction*. Using the sensing matrix A* and **Y** to reconstruct the secret image through some reconstruction algorithms in CS and obtain the sparse matrix **X**.
6. *Anti-sparsification*. Compute the secret image **I** = **Φ**^*T*^**XΦ**, where **Φ** is the orthogonal DWT matrix.

### 3.3 Theoretical results

**Theorem 1** *Suppose that the original shared image* $I\in R^{N\times N}$ *is converted into an s-sparse matrix* **X** *under DWT and the control parameter in our scheme is λ. Then the size of each shadow* **I**_*i*_ *is not smaller than O*(*s* log *N/s*)*/n* × *λN*.

*Proof*. From the sharing phase of our scheme, the size of **I**_*i*_ is the same as that of **Z**_*i*_, i.e., *M*/*n* × *λN*. Lemma 2 shows that in order to reconstruct the original image, CS requires that the number of measurements *M* does not less than *O*(*s* log* N*/*s*) [28]. Thus, the size of each shadow is not smaller than *O*(*s* log* N*/*s*)/*n* × *λN*. Note that in order to reduce the size of **I**_*i*_, the dealer can set *M* = *O*(*s* log* N*/*s*) in the secret image sharing phase.

**Theorem 2** *Suppose that the original shared image* $I\in R^{N\times N}$ *is converted into an s-sparse matrix* **X** *under DWT. If* $k\geq \frac{2ns}{O(s\log N/s)}$, *then our proposed scheme is a* (*k*, *n*)-*SSIS scheme*.

*Proof*. If all the *n* participants want to participate in the reconstruction phase, then the secret image **I** evidently can be reconstructed. If *k* (*k* < *n*) participants want to reconstruct it, then the size of formed matrix **Y** is not smaller than *kO*(*s* log *N*/*s*)/*n* × λ*N*. Lemma 1 indicates that the sensing matrix **A** should satisfy spark(**A**) > 2*s* in order to guarantee the existence of unique sparse solution of Eq (1). According to the definition of the spark, it is easy to see that 2 ≤ spark(**A**) ≤ *M* + 1. Therefore, we have

$$\begin{array}{c} s\leq \frac{1}{2}M\leq \frac{kO(s\log N/s)}{2n}, \end{array}$$ (12)

i.e., $k\geq \frac{2ns}{O(s\log N/s)}$. Thus, our proposed scheme is a (*k*, *n*)-SIS scheme if $k\geq \frac{2ns}{O(s\log N/s)}$. Note that in CS, the only way to improve the reconstruction quality is to gather more measurements. Hence, in our scheme, the more the number of shadows, the better the reconstruction quality. That is, our scheme is a (*k*, *n*)-SSIS scheme. This completes the proof.

## 4 Experimental Results and Security Analysis

In this section, we first show some experimental results to demonstrate the feasibility of our scheme, including smooth scalability, key sensitivity, noise-resilient capability and flexibility. In addition, the correlation analysis imply that our scheme can resist some statistical attacks. Last, we analyze the security of private keys in terms of outsider attacks and insider attacks.

### 4.1 Smooth scalability

**Fig 2:**
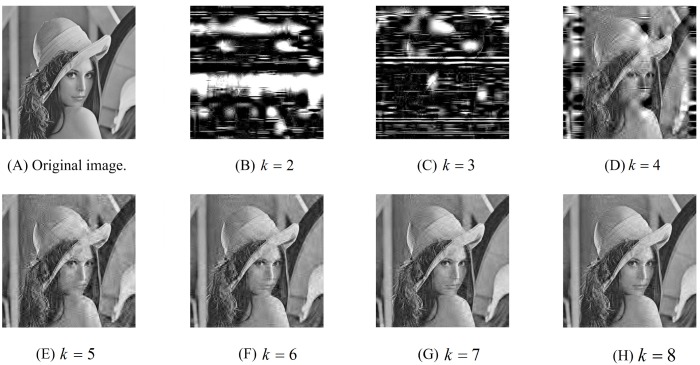
Original secret image and the reconstructed images with different threshold value *k*. (B)PSNR = 3.0434dB; (C)PSNR = 2.4072dB; (D)PSNR = 11.786dB; (E)PSNR = 24.1027dB; (F)PSNR = 25.6763dB; (G)PSNR = 26.8478dB; (H)PSNR = 27.9183dB.

Here we use the classic index, peak signal-to-noise ratio (PSNR), to evaluate the quality of the reconstructed images. Given a secret image **I** and the corresponding reconstructed image I*, the PSNR (in dB) between them is defined as $\text{PSNR}=10\log_{10}\left(\frac{MAX_{I}^{2}}{MSE_{I,I^{*}}}\right)$, where *MAX*_**I**_ and *MSE*_**I**,I*_, respectively, stand for the maximum possible pixel value of **I** and the mean square error between **I** and I*. Fig 2 shows a (4, 8)-SSIS scheme constructed by our proposed method. In our experiments, the secret image **I** is a frequently-used test image, Lena, which can be viewed as a 512 × 512 matrix. The control parameter *λ* = 0.5 and the randomly selected private keys are *k*_1_ = (0.39, 0.45), *k*_2_ = (0.78, 0.63) and *k*_3_ = (0.21, 0.97). Hence the sensing matrix $A\in R^{192\times 512}$ can be constructed using the skew tent map with the private key *k*_1_ according to the method of [28]. It is not hard to see that the size of each shadow is 24 × 256. Fig 2 indicates that if *k* < 4, then the reconstructed image is meaningless. However, when *k* ≥ 4, we can see that the larger the value of *k*, the higher the PSNR. Thus, our proposed SSIS scheme achieves the smooth scalability.

### 4.2 Key sensitivity

**Fig 3:**
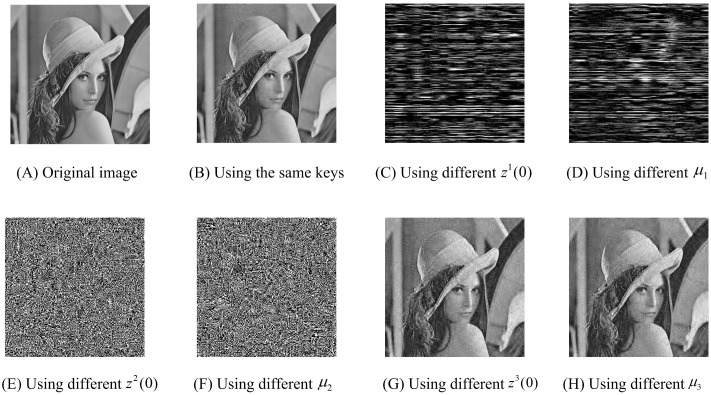
Original secret image and some reconstructed images by using different keys.

In our scheme, there exist three private keys, *k*_1_ = (*z*^1^(0), *μ*_1_), *k*_2_ = (*z*^2^(0), *μ*_2_) and *k*_3_ = (*z*^3^(0), *μ*_3_), which are used for generating sensing matrix, performing permutation and diffusion, respectively. In fact, these private keys can be viewed as six chaotic seeds in (0, 1). In these experiments, we randomly choose three private keys *k*_1_ = (0.3112, 0.5285), *k*_2_ = (0.0119, 0.3371) and *k*_3_ = (0.1622, 0.7943). And the control parameter *λ* is set to 0.5. To perform key sensitivity tests, suppose that all the *n* participants participate in the reconstruction phase, i.e., *k* = *n*, and they reconstruct the secret image using five actual chaotic seeds. They need to guess the remaining chaotic seed which they do not know. In each experiment, we also assume that the error of the remaining actual seed and the guessed seed is 10^−16^. Fig 3C–3F indicate that using a slightly changed chaotic seed in *k*_1_ or *k*_2_ results in the complete failure of the reconstruction phase. However, Fig 3G and 3H show that the secret image can be reconstructed with relatively low quality if the private key *k*_3_ is not fully matched. Even so, our proposed SSIS scheme is also practical because it is almost impossible that an adversary can correctly guess the private keys *k*_2_ and *k*_3_ at the same time. Even if the precision is 10^−16^, the probability that the adversary guess *k*_2_ and *k*_3_ correctly is 10^−64^, which is small enough to provide a strong security guarantee. That is, the key space of our scheme is large enough to make brute-force attacks infeasible.

### 4.3 Noise-resilient capability and Flexibility

**Fig 4:**
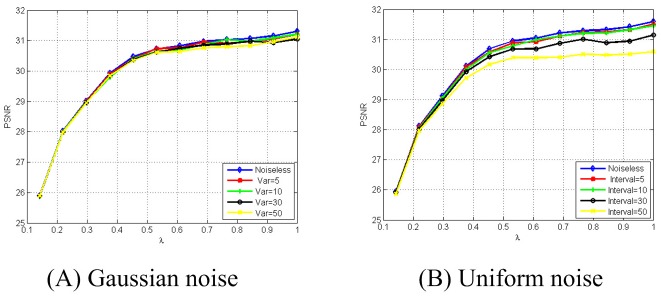
PSNR between the original image and its reconstructed image when noise exist.

Most existing (*k*, *n*)-SSIS schemes do not provide noise-resilient capability. In some Shamir-type (*k*, *n*)-SSIS schemes, it is evidently impossible to perform the reconstruction phase correctly if there exist some types of noise in the communication channel. However, in the real word, noise cannot be avoided. Thus, designing (*k*, *n*)-SSIS schemes with noise-resilient capability is an important challenge in this field. In our construction, we first use CS to compress the secret image and then cut the compressed image through a control parameter *λ*. Note that CS has strong noise-resilient capability. In our experiments, the secret image **I** is Lena and we still choose three private keys from (0, 1) at random. Suppose *n* = 10 and all the participants will reconstruct the original secret image **I** when there exist some types of noise in the communication channel. Here we consider two types of noise, Gaussian and Uniform noise. In Fig 4A, we consider Gaussian noise with *mean* = 0 and different *variance*, i.e., 5, 10, 30, and 50. In Fig 4B, we consider Uniform noise with different *interval*, i.e., [−5, 5], [−10, 10], [−30, 30], and [−50, 50]. Experimental results reveal that the effects of Gaussian and Uniform noise on the PSNR is inappreciable. In addition, it is not difficult to find that when *λ* > 0.5, the PSNR is almost unchangeable. This fact provides an experimental evidence for our previous claim (Remark 2), i.e., the most of the original image information are concentrated on the left half of the image after CS. Thus, in practice, the dealer can select a optimal parameter *λ* ≈ 0.5 to achieve a compromise between the size of each shadow and the quality of the reconstructed image.

### 4.4 Correlation analysis

**Fig 5:**
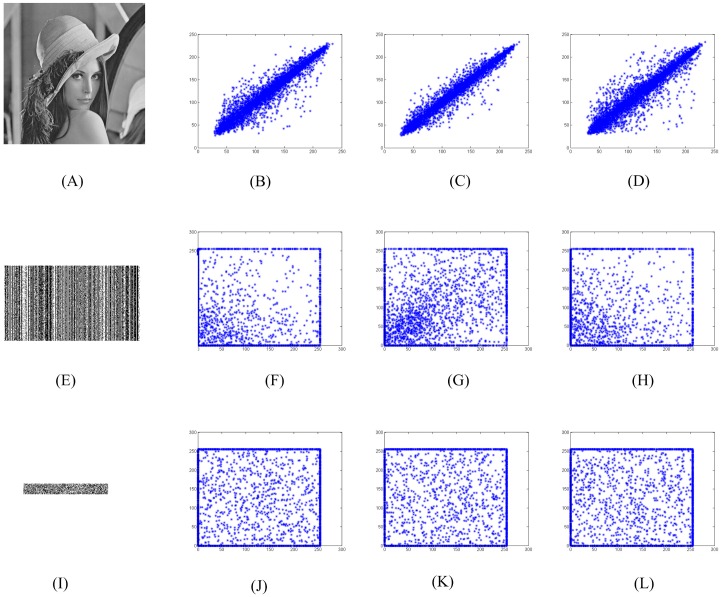
Correlations between two adjacent pixels in the horizontal, vertical and diagonal directions.

For most nature images, the adjacent pixels are highly correlated to each other. To resist some statistical attacks, it requires that the correlation between two adjacent pixels in the shadows should be low enough. For a given image, the correlation coefficient of *K* pairs of adjacent pixels is defined as

$$\begin{array}{c} C=\frac{\sum_{i=1}^{K}\left(x_{i}-E(x))(y_{i}-E(y)\right)}{\sqrt{\left(\sum_{i=1}^{K}{\left(x_{i}-E(x)\right)}^{2})(\sum_{i=1}^{K}{\left(y_{i}-E(y)\right)}^{2}\right)}}, \end{array}$$ (13)

where ${\left\{x_{i}\right\}}_{i=1}^{K}$ and ${\left\{y_{i}\right\}}_{i=1}^{K}$ denote the corresponding grey values that we selected randomly in the image, $E(x)=\frac{1}{K}\sum_{i=1}^{K}x_{i}$ and $E(y)=\frac{1}{K}\sum_{i=1}^{K}y_{i}$. To form the final *n* shadows {**I**_*i*_}_*i* ∈ [*n*]_, the original secret image **I** undergos seven steps in our sharing phase, such as *Sparsification* and *Compression*. Here the compressed image and the final shadows are referred to the formed image after *Compression* and *Diffusion*, respectively. To measure the correlations between two adjacent pixels in the horizontal, vertical and diagonal directions, 8000 pairs of adjacent pixels are selected randomly from the secret image, the compressed image and a final shadow. Fig 5A, 5E and 5I respectively show the original image, the compressed image and a final shadow. It indicates that the nonlinear operations in our scheme are necessary for resisting statistical analysis. Fig 5B–5D show the correlations of the original image in the horizontal, vertical and diagonal directions, respectively. Similarly, Fig 5F–5H and 5J–5L show the correlations of compressed image and a final shadow in these three different directions, respectively. The experimental results show that the correlations in the horizontal and diagonal directions are greatly decreased after *Compression*. However, the correlations in the vertical is also very high. Though the nonlinear operation *Diffusion*, the final shadow has very low correlation in these three directions. The same result can be seen from Table 1.

**Table 1:** Correlation coefficients of adjacent pixels.

Correlation coefficient	Horizontal	Vertical	Diagonal
Original image	0.9740	0.9848	0.9576
Compressed image	0.0567	0.7112	0.1128
Final shadow	0.0033	0.0192	0.0191

### 4.5 Security analysis

The security is a critical issue that we should consider in our SSIS scheme. There are *n* participants in total and there exist two types of attacks, namely, outsider attacks (attacks from outside parties) and insider attacks (attacks from insider participants). No matter what type of attack might be involved, we assume that the attacker knows everything about our SIS system except the private keys because of Kerckhoffs’s principle [31].

For an outsider attacker, he does not know the three private keys *k*_1_, *k*_2_ and *k*_3_. The key sensitivity texts in Section 4.2 indicate that the key space is large enough to make brute-force attacks infeasible. Next, we will analyze our scheme from a cryptographic perspective, including ciphertext-only attack and known-plaintext attack. Ciphertext-only attack means that the attacker is assumed to have access only to a set of ciphertexts. Known-plaintext attack refers to an attack model for cryptanalysis where the attacker has access to both the plaintext and its corresponding ciphertext. In fact, Rachlin and Baron [32] proved that CS can provide a computational guarantee of secrecy and there does not exist an attacker who can reconstruct the original signal successfully under the model of ciphertext-only attack. Recently, Cambareri et al. [33] introduced a quantitative analysis to compressed sensing-based cryptosystems against know-plaintext attack. These previous studies on the security of CS model provide sufficient evidence to demonstrate that the *Sparsification* and *Compression* steps in our scheme can resist ciphertext-only attack and known-plaintext attack [32–34]. In addition, the *Permutation* and *Diffusion* steps also provide a more reliable guarantee for the security of our scheme.

However, if there exists a malicious insider participant *P*_i*_, our original (*k*, *n*)-SSIS scheme (in Section 3) has a vulnerability because each participant has the same private keys. As long as *P*_i*_ obtains arbitrary other *k* − 1 image shadows in some way, such as eavesdropping on the communication channel and attacking the storage systems of other participants, he can reconstruct the secret image by himself. For this security issue, a natural solution is to transmit each shadow over a secure anti-eavesdropping communication channel and improve the security of storage systems of all participants. Obviously, this prevention method is not the best choice because of huge cost. In face of an insider attacker *P*_i*_ who possesses valid chaotic keys, we provide an alternative scheme which is a slightly variant of our original scheme to overcome this deficiency. The variant is the same as our original scheme except the key distribution mechanism. In the key distribution mechanism of our variant, the dealer first chooses a random real value *k*_1_ ∈ (0, 1) and produces *n* shadows {*k*_1*i*_}_*i* ∈ [*n*]_ using Shamir’s (*k*, *n*)-SS scheme. And then he chooses 2*n* different values {*k*_2*i*_}_*i* ∈ [*n*]_ and {*k*_3*i*_}_*i* ∈ [*n*]_ at random. Last, the dealer transmits the private key {*k*_1*i*_, *k*_2*i*_, *k*_3*i*_} to each participant *P*_*i*_. In the image sharing phase, the dealer uses the key *k*_1_ to produce the sensing matrix and *k*_2*i*_, *k*_3*i*_ to perform permutation and diffusion of *P*_*i*_. In the image reconstruction phase, any *k* participants can recover *k*_1_ using Lagrange interpolation and then produce the sensing matrix. The *Reverse operations* can also be evidently performed because each participant knows his private key. In this variant, the malicious attacker *P*_i*_ only knows his own private key and does not know the private keys of the other participants. In fact, *P*_i*_ becomes an “outsider attacker” with respect to the other participants. Thus, our proposed variant with different key distribution mechanism can effectively overcome some insider attacks.

## 5 Comparison

**Fig 6:**
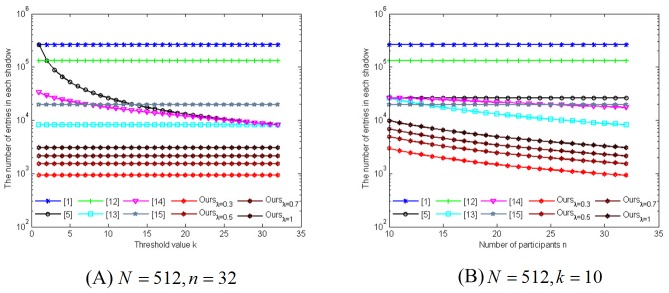
Comparison of shadow sizes between our scheme and some existing SIS schemes.

In this section, we provide a comparison between our scheme and some typical existing SIS schemes [1, 5, 12–15] in terms of shadow size, scalability, noise-resilient capability and flexibility, respectively. We assume that the size of the shared image is *N* × *N*. Note that the size of each shadow in our scheme is *M*/*n* × *λN*, where *M* < *N* and *λ* ∈ (0, 1]. Liu et al. proposed an SIS scheme with small shadows and noise-resilient capability [15], and the parameter *γ* in Table 2 is the compression ratio of entropy coding in the secret image sharing phase (see Step 4 of [15]). Table 2 shows the overall results and Fig 6 specifically considers the comparison of shadow sizes under a reasonable parameter setting. Table 2 indicates the superiority of our scheme. That is, our scheme has some new functionality, such as noise-resilient capability and flexibility, which some previous SIS schemes do not possess. Evidently, we can see that the shadow size of our scheme is much smaller than those of the aforementioned schemes. Here we also provide further evidence from an experimental point of view. In Fig 6, the size of secret image *N* × *N* is 512 × 512 and the number of measurements *M* in CS is 192. To make the shadow size in [15] as small as possible, we set the parameter *γ* equal to 5. In Fig 6A, the number of all participants *n* is 32 and we investigate the number of entries in each shadow with the increasing of the threshold value *k*. Note that such number plays a crucial role in the communication and storage overhead. Similarly, Fig 6B investigates the number of entries in each shadow in terms of *n* when the threshold value *k* is 10. The experimental results show that no matter how large *λ* is, the number of entries in each shadow in our scheme is very small compared to those in the other schemes. Thus we can conclude that our proposed scheme is competitive compared to these typical existing SIS schemes.

**Table 2:** Comparison between our scheme and some typical (*k*, *n*)-SIS schemes.

Scheme	Shadow size	Scalability	Smooth scalability	Noise-resilient	Flexibility
[1]	*N* × *N*	No	No	No	No
[5]	*N* × *N*/*k*	No	No	No	No
[12]	*N* × *N*/2	Yes	No	No	No
[13]	*N* × *N*/*n*	Yes	No	No	No
[14]	$N\times N(1+\sum_{i=k+1}^{n}1/i)/n$	Yes	Yes	No	No
[15]	*M* × *N*/*γ*	No	No	Yes	No
Ours	*M*/*n* × *λN*	Yes	Yes	Yes	Yes

## 6 Conclusion

Based on CS, a secure and efficient (*k*, *n*)-SSIS scheme with flexible shadow sizes is present in this paper. The main idea behind our construction is to reduce the size of each shadow through two procedures, i.e., *Compression* and *Downsizing*. And then the dealer performs two nonlinear operations, *Permutation* and *Diffusion*, for improving security. Thus, the dealer can achieve a compromise between the size of each shadow and the quality of the reconstructed image. Compared to some existing SSIS schemes, the experimental results and security analysis indicate the infeasibility and potential superiority of our scheme.
